# The Incidence and Severity of Patient-Reported Side Effects of Chemotherapy in Routine Clinical Care: A Prospective Observational Study

**DOI:** 10.7759/cureus.38301

**Published:** 2023-04-29

**Authors:** Bhavana Katta, Chellappa Vijayakumar, Souradeep Dutta, Biswajit Dubashi, Vishnu Prasad Nelamangala Ramakrishnaiah

**Affiliations:** 1 Surgery, Jawaharlal Institute of Postgraduate Medical Education and Research, Puducherry, IND; 2 Medical Oncology, Jawaharlal Institute of Postgraduate Medical Education and Research, Puducherry, IND

**Keywords:** compliance, self-reported, quality of life, side effects, cancer chemotherapy

## Abstract

Introduction: Understanding patients' self-reported chemotherapy side effects is significant because it affects patients' quality of life (QOL) and compliance with treatment. Our current knowledge of chemotherapy side effects comes from available literature, whose external validity is questionable. Moreover, there are very few studies available in the literature that focus on various cancers and their associated side effects.

Methods: A single-center, prospective observational study was conducted at a tertiary care center from July 2019 to July 2021. After deriving the sample size, we interviewed 76 consecutive study patients with gastric, periampullary, colorectal, and breast cancer for six months after chemotherapy initiation with a structured patient-reported outcome tool adapted in English and Tamil to record the side effects like diarrhea, vomiting, chest pain, constipation, dyspnea, fatigue, mucositis, and rash. The grading of symptoms was done according to the Common Terminology Criteria for Adverse Events version 5.0. The frequency and prevalence of side effects were calculated as the number of patients who reported the side effect of any grade at least once during the follow-up period. The incidence rate of side effects was calculated in terms of person-time. The association between each side effect and cancer type was calculated using the chi-square test and Fisher's exact test as appropriate.

Results: Of the 77 patients in the study, 51.9% were male, 63.6% were between 40 and 60 years of age, 45.5% had stage-3 disease, and 44.2% received neoadjuvant treatment. During the six-month follow-up period, 97.4% of patients experienced at least one side effect. Fatigue was the most common side effect (87%), followed by loss of appetite (71.4%) and diarrhea (49.4%). Approximately 66.7% of patients experienced six or more side effects. There was a statistically significant difference in the frequency of side effects between cancer types. However, age, socioeconomic status, BMI, comorbidity, chemo-intent, and stage of disease did not affect the frequency of side effects.

Conclusions: This study highlights the need to integrate patient-reported side effects into routine clinical practice. Identifying these side effects, even if they are mild in intensity, and managing them in a timely manner may improve the patient's emotional state, QOL, and compliance with chemotherapy.

## Introduction

The incidence of cancer is increasing globally, with an estimated 19.3 million new cases worldwide in 2020 [1]. In India, the cancer burden for 2020 was estimated to be 98.7 per 100,000 population, accounting for 1,392,179 patients [2]. Improved treatment modalities and increased overall survival have resulted in an increase in the number of patients living with cancer [3]. However, chemotherapy drugs that are effective in killing cancer cells can also damage normal cells and cause side effects [4], which may affect patients' quality of life (QOL) and psychosocial well-being [5].

While clinical trials provide important information about chemotherapy side effects, their results may not be generalizable to routine clinical care. Patients in clinical practice may experience more chemotherapy side effects than reported in clinical trials which often do not accompany appropriate external validation [6]. For instance, a clinical trial reported a frequency of 34% and 45% for diarrhea (any grade) in patients receiving three months and six months of fluorouracil-/oxaliplatin-/leucovorin- or capecitabine-/oxaliplatin-based chemotherapy, respectively [7]. An observational study on chemotherapy side effects done in clinical practice showed that the frequency of diarrhea (any grade) in colorectal cancer is 75% (107 of 142) [8]. In clinical trials, chemotherapy side effects are often reported by clinicians, who may underestimate the incidence and severity of side effects compared to patient reports [9]. To obtain more generalizable results, observational studies that rely on patient-reported outcomes in routine clinical care are necessary [10]. Although there are some observational studies available in the literature on patient-reported chemotherapy side effects for specific cancer types, stages, chemotherapy regimens, or side effects [11,12], there are few studies available on the side effects of chemotherapy across various cancers and treatment regimens [13]. Moreover, no established research is available on the Indian population.

Therefore, this study aims to estimate patient-reported chemotherapy side effects, including diarrhea, vomiting, chest pain, constipation, dyspnea, fatigue, mucositis, and rash in patients with gastric, periampullary, colorectal, and breast cancers in routine clinical care in the Indian population rather than in clinical trials. Identifying and managing these side effects, even if they are mild, can improve patients' emotional state, quality of life, and compliance with chemotherapy.

## Materials and methods

This study was a single-center, prospective observational study done in the Departments of Surgery and Medical Oncology at the Jawaharlal Institute of Postgraduate Medical Education and Research, Puducherry, which is a tertiary care teaching hospital in South India. The study's ethical clearance was obtained from the Institute Ethics Committee (approval number: JIP/IEC/2019/0202). The study period was two years, from July 2019 to July 2021. All the consecutive patients with gastric, periampullary, colorectal, and breast cancers above 18 years of age and receiving chemotherapy were included in the study. Patients not willing to consent or participate in clinical trials are excluded from the study. The primary objective of this study is to assess the incidence and severity of patient-reported chemotherapy side effects in gastric, periampullary, colorectal, and breast cancers in routine clinical care during the follow-up period of six months.

Study patients

Informed consent was obtained from patients who were included in the study. Demographic data like age, sex, phone number, address, BMI, education, income, occupation, comorbidities, type and staging of carcinoma, chemotherapy intent, chemotherapy regimen, imaging, histopathology, and any hospital admissions were collected from hospital medical records. The socioeconomic status of the participants was calculated using a modified Kuppusamy scale [14].

Study methods

Eligible patients were identified and informed about the study and the need for monthly face-to-face or telephone interviews for six months. Patients who gave consent were included in the study and interviewed for six months after chemotherapy initiation. The first interview was three to four days after the first cycle of chemotherapy and then every month for the next five months. (Due to the COVID-19 pandemic, most of the subsequent interviews were via telephone.) Participants were asked with a structured set of questions if they had experienced any side effects like diarrhea, vomiting, chest pain, constipation, dyspnea, fatigue, mucositis, and rash, with examples of each grade. These side effects were selected because they are common side effects observed in patients receiving chemotherapy and can be easily expressed from the patient's perspective. This patient-reported outcome tool was made according to the Common Terminology Criteria for Adverse Events version 5.0 and was adapted into English and Tamil.

Sample size calculation

Sample size calculation was performed using OpenEpi v3.03, with a calculated sample size of 63, a power of 80%, relative precision of 10%, and CI of 95%, based on an overall adverse effects rate of 86% [8]. With an attrition rate of 20%, the final sample size for the study was 76. A convenient sampling technique was used.

Statistical analysis

The statistical analysis was performed using SPSS Statistics version 21.0 (IBM Corp. Released 2012. IBM SPSS Statistics for Windows, Version 21.0. Armonk, NY: IBM Corp.). The frequency and prevalence of side effects were calculated as the number of patients who reported the side effect of any grade at least once during the follow-up period. The incidence rate of side effects was calculated in terms of person-time. Once individuals experienced the selected side effect, they were censored. The severity of side effects was determined using the worst grade of each side effect experienced by the individual during follow-up. Data were presented as percentages for categorical variables. The association between each side effect and cancer type was calculated using the chi-square test and Fisher's exact test as appropriate, with a p-value of less than 0.05 considered significant.

## Results

Demographic and clinical data

A total of 77 patients were included in the study. The majority of 34% were breast cancer patients, and the least were periampullary cancer (4%) patients. In the study cohort, 48% were females, and most patients were between 40 and 60 years of age (63.6%). About 58.4% of the cohort had BMI ≥ 25 kg/m^2^, and 33.8% of the cohorts in the study had comorbidities (diabetes mellitus (19.5%)/hypertension (24.7%)/coronary artery disease (3.9%)/others (6.5%)). About 45% of patients had locally advanced disease, 14% had metastatic disease, and 44.2% received neoadjuvant chemotherapy (Table 1).

**Table 1:** Demographic and clinical data of the study SES: socioeconomic status, LM: lower middle, UM: upper middle, DM: diabetes mellitus, CAD: coronary artery disease, HTN: hypertension, BMI: body mass index

S.No	Variables		Carcinoma				
			Breast (n=26)	Stomach (n=23)	Colorectal (n=25)	Periampullary (n=3)	Total (n=77)
			N %	N %	N %	N %	N %
1	Sex	Female	26(100)	5(21.7)	6(24)	0	37(48.1)
		Male	0	18(78.3)	19(76)	3(100)	40(51.9)
2	Age group	<40	4(15.4)	3(13)	5(20)	0	12(15.6)
		40-60	19(73.1)	14(60.9)	13(52)	3(100)	49(63.6)
		>60	3(11.5)	6(26.1)	7(28)	0	16(20.8)
3	SES	UM	20(76.9)	7(30.4)	13(52)	1(33.3)	41(53.2)
		LM	6(23.1)	16(69.6)	12(48)	2(66.7)	36(46.8)
4	BMI	<18.5	0	0	1(4)	0	1(1.3)
		18.5-24.9	8(30.8)	11(47.8)	11(44)	1(33.3)	31(40.3)
		>/=25	18(69.2)	12(52.2)	13(52)	2(66.7)	45(58.4)
5	Comorbidity	Yes	10(38.5)	7(30.4)	8(32)	1(33.3)	26(33.8)
		No	16(61.5)	16(69.6)	17(68)	2(66.7)	51(66.2)
6	DM	Yes	4(15.4)	4(17.4)	7(28)	0	15(19.5)
		No	22(84.6)	19(82.6)	18(72)	3(100)	62(80.5)
7	HTN	Yes	5(19.2)	7(30.4)	6(24)	1(33.3)	19(24.7)
		No	21(80.8)	16(69.6)	19(76)	2(66.7)	58(75.3)
8	CAD	Yes	2(7.7)	1(4.3)	0	0	3(3.9)
		No	24(92.3)	22(95.7)	25(100)	3(100)	74(96.1)
9	Others	Yes	3(11.5)	0	2(8)	0	5(6.5)
		No	23(88.5)	23(100)	23(92)	3(100)	72(93.5)
10	Staging	1	0	0	0	2(66.7)	2(2.6)
		2	11(42.3)	10(43.5)	8(32)	0	29(37.7)
		3	15(57.7)	5(21.7)	14(56)	1(33.3)	35(45.5)
		4	0	8(34.8)	3(12)	0	11(14.3)
11	Chemo intent	Adjuvant	7(26.9)	3(13)	20(80)	3(100)	33(42.9)
		Neo-adjuvant	19(73.1)	13(56.5)	2(8)	0	34(44.2)
		Palliative	0	7(30.4)	3(12)	0	10(13)

Frequency of patient-reported side effects

In the study, 97.4 % (n=75) of patients reported at least one side effect during the follow-up period; this was similar across cancers. Fatigue was the most common side effect reported overall 87% (n= 67), followed by loss of appetite 71.4% (n=55), diarrhea 49.4% (n=38), nausea 32.5% (n=25), and dyspnea 32.5% (n=25). There was a statistically significant difference in the frequency of side effects between cancer types for diarrhea (p-value 0.002), constipation (p-value 0.016), vomiting (p-value <0.001), nausea (p-value 0.002), mucositis (p-value <0.001), and dyspnea (p-value <0.001) (Table 2).

**Table 2:** Frequency of patient-reported side effects by cancer type in the study cohort *p-value calculated for each side effect by cancer type using the chi-squared test

S.No	Side effects	Carcinoma					p-value*
		Breast (n=26)	Stomach (n=23)	Colorectal (n=25)	Periampullary (n=3)	Overall (n=77)	
		N (%)	N (%)	N (%)	N (%)	N (%)	
1	Any side effects	25(96.2)	23(100)	24(96)	3(100)	75(97.4)	0.790
2	Chest pain	1(3.8)	1(4.3)	0	0	2(2.6)	0.756
3	Fatigue	25(96.2)	19(82.6)	20(80)	3(100)	67(87)	0.278
4	Diarrhea	5(19.2)	13(56.5)	18(72)	2(66.7)	38(49.4)	0.002
5	Constipation	0	7(30.4)	7(28)	0	14(18.2)	0.016
6	Vomiting	0	10(43.5)	4(16)	3(100)	17(22.1)	<0.001
7	Loss of appetite	17(65.4)	17(73.9)	19(76)	2(66.7)	55(71.4)	0.844
8	Nausea	6(23.1)	12(52.2)	4(16)	3(100)	25(32.5)	0.002
9	Mucositis	0	7(30.4)	14(56)	1(33.3)	22(28.6)	<0.001
10	Dyspnoea	12(46.2)	10(43.5)	0	3(100)	25(32.5)	<0.001
11	Rash	0	0	3(12)	0	3(3.9)	0.090

Incidence of patient-reported side effects

Fatigue had an overall high incidence in the study cohort (25.9 per 100 person-month), followed by loss of appetite (17.7 per 100 person-month). Similarly, chest pain had a low incidence in the study cohort (0.4 per 100 person-month), followed by a rash (0.7 per 100 person-month) (Table 3).

**Table 3:** Incidence of patient-reported side effects by cancer type during the study period

S.No	Side effect	Carcinoma				
		Breast (n=26)	Stomach (n=23)	Colorectal (n=25)	Periampullary (n=3)	Overall (n=77)
		Incidence %	Incidence %	Incidence %	Incidence %	Incidence %
1	Chest pain	0.7%	0.7%	0.0%	0.0%	0.4%
2	Fatigue	27.3%	22.1%	23.8%	42.9%	25.9%
3	Diarrhea	3.5%	13.3%	18.9%	18.2%	11.0%
4	Constipation	0.0%	5.7%	5.2%	0.0%	3.3%
5	Vomiting	0.0%	9.6%	2.9%	33.3%	4.2%
6	Loss of appetite	14.8%	17.5%	22.1%	16.7%	17.7%
7	Nausea	4.2%	11.1%	2.9%	25.0%	6.2%
8	Mucositis	0.0%	6.3%	12.5%	5.9%	5.4%
9	Dyspnea	8.3%	8.5%	0.0%	33.3%	5.9%
10	Rash	0.0%	0.0%	2.1%	0.0%	0.7%

Severity of patient-reported side effects

For 96.1% of study participants, the overall grade of side effects experienced was grade I or II, and 2.6% of participants reported grade III side effects (chest pain and dyspnea) (Table 4).

**Table 4:** Severity of patient-reported side effects during the study period

S.No	Variables	None N(%)	Grade 1 N(%)	Grade 2 N(%)	Grade 3 N(%)
1	Chest pain	75(97.4)	1(1.3)	0	1(1.3)
2	Fatigue	10(13)	33(42.9)	34(44.2)	0
3	Diarrhea	39(50.6)	20(26)	18(23.4)	0
4	Constipation	63(81.8)	7(9.1)	7(9.1)	0
5	Vomiting	60(77.9)	5(6.5)	12(15.6)	0
6	Loss of appetite	21(27.3)	25(32.5)	31(40.3)	0
7	Nausea	52(67.5)	14(18.2)	11(14.3)	0
8	Mucositis	55(71.4)	10(13)	12(15.6)	0
9	Dyspnea	52(67.5)	19(24.7)	5(6.5)	1(1.3)
10	Rash	74(96.1)	3(3.9)	0	0

## Discussion

In recent decades, there has been a rise in cancer incidence. Chemotherapy, along with surgery and radiotherapy, is an indispensable modality in cancer treatment. Thanks to the effective chemotherapy drugs available in the last few decades, overall survival has increased. As the overall survival of cancer patients has improved, knowledge about chemotherapy side effects affecting their QOL and compliance to treatment has become more critical. Currently, the available knowledge about chemotherapy side effects comes from clinical trials. However, their external validity is questionable, and there are few observational studies across various cancers, regimens, and side effects. In a recent prospective study among breast cancer patients by Galizia et al., it was concluded that doctors often underestimate the incidence and severity of treatment-related side effects in clinical practice, and the extent of underreporting is more significant in high-volume centers. The study also found that patients' questionnaires were a reliable tool for collecting side effect-related information [15].

Frequency of patient-reported side effects

The frequency of any side effect reported by patients in this study is 97.4%, with fatigue being the most common side effect reported (87%). The study results are similar to a cross-sectional survey conducted in the United States by Henry et al., which showed that 88% of participants reported at least one side effect during their cancer treatment, and fatigue was the most common side effect reported (80%) [13]. Another study by Alison Pearce et al. about patient-reported side effects in chemotherapy showed that 86% of participants reported at least one side effect during the study period, and fatigue was the most common side effect reported (85%), consistent with the findings in this study [8].

The frequency of side effects in this study was comparable to previous observational studies available in the literature. The increased frequency of side effects reported by patients in this study shows that chemotherapy side effects are more common in clinical practice than in clinical trials.

Incidence of patient-reported side effects

It is not easy to compare the incidence rates of each side effect with available studies in the literature because the follow-up period differs between studies. In a similar study done in the Australian population, the incidence rate of any event was 0.22 events per person per month of follow-up, whereas, in this study, the incidence rate of any event was 0.65 events per person per month of follow-up [8]. The difference in the incidence rates reported may be due to data collection methods like providing an example of each side effect and its grade helps in a better estimate of side effects than open-ended questions. The difference might also be due to the time of data collection; for example, immediately after chemotherapy, patients might report more side effects than those collected after a few days of chemotherapy. Additionally, due to the ongoing COVID-19 pandemic, many patients with side effects might be left untreated due to difficulty in accessing healthcare, and ongoing side effects reported in monthly interviews could have also caused the higher incidence rates in the study.

Severity of patient-reported side effects

Most patients in the study reported mild (grade I and II) side effects during the six-month follow-up period (96.1%). The study by Alison Pearce et al. about patient-reported side effects in chemotherapy showed that 35% of participants reported moderate (grade III) side effects, and 27% reported severe (grade IV) side effects during the median 5.64 months of follow-up [8].

The discrepancy in the severity of side effects reported between studies can be ascertained by (a) sample size (77 vs. 441), (b) age group of the study participants (in this study, 63.6% of participants are between 40 and 60 years of age, whereas in the other study, 27.5% of participants are above 65 years of age), (c) type of cancer patients included in the study (present study included gastric, periampullary, colorectal, and breast cancer patients. whereas the other study included breast, colorectal, and lung cancer patients), (d) difference in the stage of the disease included in the studies (in the current study, 14.3% of participants had stage 4 disease, whereas the previously said study had 52.4% of participants with stage 4 disease), (e) and many patients did not receive their intended chemo cycles as planned due to the COVID-19 pandemic; this delay between chemo cycles could have influenced the severity of side effects in the study [8]. However, both studies showed that many patients reported ongoing side effects during their chemotherapy. Even though mild, persistent symptoms can affect the patient's emotional state, which further influences compliance to treatment and causes them to delay the next chemo cycle or miss the chemotherapy cycles. Some patients reported side effects after a few months of treatment, and a few reported severe side effects during the follow-up period because of which their treatment regimen was changed; this shows the importance of monitoring side effects in patients receiving chemotherapy.

Limitations

Dropout rates and patient compliance with chemotherapy could not be evaluated in this study, and the overall sample size was relatively small. Side effects of chemotherapy could not be monitored for the entire treatment duration and follow-up. Only breast, gastric, periampullary, and colorectal cancers were included in the study. The study did not record the proportion of patients who received treatment for side effects. Recall bias happened due to monthly interviews. There is no control group to compare; hence, it was difficult to determine the proportion of reported side effects due to chemotherapy.

## Conclusions

This study shows the need to integrate patient-reported outcomes into routine clinical care. Identifying these common chemotherapy side effects in clinical practice and their in-time management can help increase patient compliance and thus decrease dropout rates. It may help to improve the patient's emotional state and QOL.
